# Polder Effects on Sediment-to-Soil Conversion: Water Table, Residual Available Water Capacity, and Salt Stress Interdependence

**DOI:** 10.1155/2013/451710

**Published:** 2013-08-05

**Authors:** Raymond Tojo Radimy, Patrick Dudoignon, Jean Michel Hillaireau, Elise Deboute

**Affiliations:** ^1^IC2MP-HydrASA Laboratory UMR 7285, ENSIP, 40 Avenue du Recteur Pineau, 86022 Poitiers, France; ^2^INRA Domaine Expérimental Saint Laurent-de-la-Prée, 17450 Saint Laurent-de-la-Prée, France

## Abstract

The French Atlantic marshlands, reclaimed since the Middle Age, have been successively used for extensive grazing and more recently for cereal cultivation from 1970. The soils have acquired specific properties which have been induced by the successive reclaiming and drainage works and by the response of the clay dominant primary sediments, that is, structure, moisture, and salinity profiles. Based on the whole survey of the Marais Poitevin and Marais de Rochefort and in order to explain the mechanisms of marsh soil behavior, the work focuses on two typical spots: an undrained grassland since at least 1964 and a drained cereal cultivated field. The structure-hydromechanical profiles relationships have been established thanks to the clay matrix shrinkage curve. They are confronted to the hydraulic functioning including the fresh-to-salt water transfers and to the recording of tensiometer profiles. The CE_1/5_ profiles supply the water geochemical and geophysical data by their better accuracy. Associated to the available water capacity calculation they allow the representation of the parallel evolution of the residual available water capacity profiles and salinity profiles according to the plant growing and rooting from the mesophile systems of grassland to the hygrophile systems of drained fields.

## 1. Introduction

The degradation and restoration of wetlands are consequences and/or objectives of the successive human activities which are, or which have been, governed by the society needs. The coastal marshes have often been the result of land reclaiming from the sea by successive polders. The initial attempt was to earn new areas, generally fit for farming from primary muddy sediments. The main consequences due to the polder mechanisms are to provoke the “primary” muddy sediment-to-soil evolution by desiccation, consolidation, and maturation of the clay-rich material. The final objective was the development of “dry” territories. The mechanism is generally associated to the descending of the water table levels. In case of too slow desiccation rates, the phenomenon has been emphasized by drainage. On the hydraulically point of view, the need of restoration is the consequence of the too large extension of the “dry marshes” in place of the “wet marshes” which constitute the residual and “primitive” domains of the flora and wildlife. In these conditions the control of the water table levels can be a cause of fighting due to the different objectives and economical or ecological interests.

One of the objectives prevailing during the years 1970–1980 was the conversion of the grazing system to the cereal cultivation system. Unfortunately the too high water table levels limit the rooting depth for cereals. Consequently, the drainage was systematically developed during these years [1]. Besides, a consequence of the cereal system development was the impact of the fertilizers and phytosanitaries on the coastal oyster and mussel cultures [2]. Thus the cultivation system approach has to consider the exploitation-environment relationship since the 1970s [3]. In fact, the functioning of the coastal marsh soils is mainly based on their salinity and sodicity evolutions. These two factors depend on the water table levels which are governed by the successive polders since the Middle Ages and more recently by the drainage. However, the drainage efficiency may be limited because of the soil structural stability and low permeability in such clay dominant soils [1, 4–6]. Due to this problem, successive typologies of soil exploitation with and without drainage were proposed for these territories: that is, stable, intermediate, and unstable “dry marshes” [7]. Finally, Pons and Gerbaud propose in 2005 [6] a soil classification based on the salinity, sodicity, and a dispersion index measurement derived from the Emerson method [6, 8].

**Figure 1:**
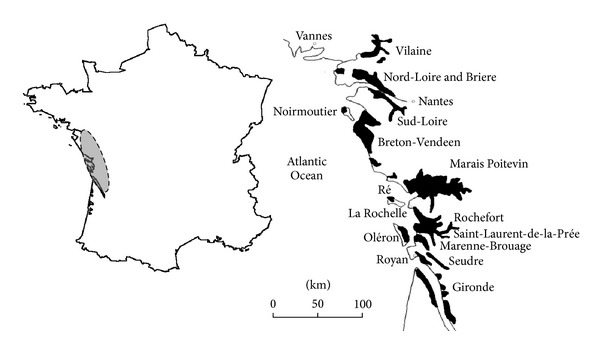
Location of the “Marais de Rochefort” and Marais Poitevin on the West Atlantic coast of France. The INRA experimental site of St Laurent de la Prée is located in the “Marais de Rochefort.”

**Figure 2:**
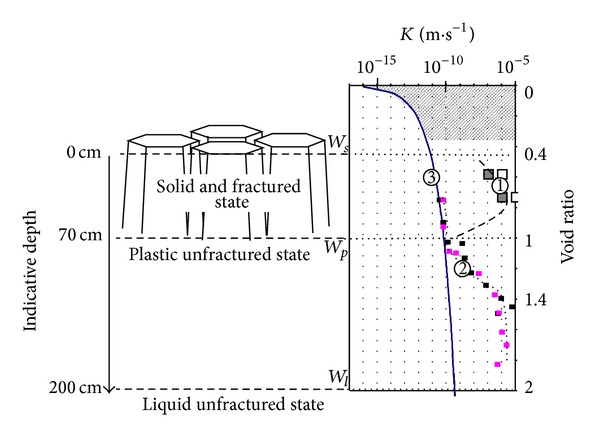
Parallel representation of the vertical evolution of the soil structure and hydraulic conductivity. In the *W*_*s*_-*W*_*p*_surface layer the clay dominant soil evolves in its solid state. In the *W*_*p*_-*W*_*l*_ subjacent layer the clay matrix evolves in plastic-to-liquid states. The hydraulic conductivity has been “*in situ*” measured by infiltrometry in the fractured surface layer (large squares), via oedometer compressibility tests on saturated and consolidated clay material (small squares), and calculated taking into account the clay matrix microstructure parameters (continuous line; [18]). The dashed domain does not exist “*in situ*” (from Gallier et al. [15]).

Eventually, the two main problems induced by the land reclamation on sea wetlands are the drastic structure evolution of the clay dominant sediments due to the desiccation and shrinkage phenomena and the induced soil salinity and sodicity evolutions. The consequences are the superimposition of the water and salt stresses due to the plant growing. The present paper is based on works made on the French Atlantic coast wetlands many years ago [9–15]. The successive works focused on the relationships which can exist between the microstructure evolution of clay matrices and the macroscopic properties of the clay dominant soils. The coastal territories were chosen thanks to their textural and mineralogical homogeneity of soils and sediments and thanks to the large water content domain available along each vertical profile. The primary investigations were driven in order to ensure the homogeneity of the shallow sedimentary formations throughout the marshlands of the Marais Poitevin and Marais de Rochefort. Secondly, the investigations were concentrated on the INRA experimental site of St Laurent de la Prée for all the calibrations of soil structure-hydromechanical properties relationships [13, 15] (Figures 1 and 2). Thirdly the goal was to understand the role of the soil structure evolution on the plant growing and crop yield. The work is based on the study of the soil profiles developed on drained cereal systems and on undrained grasslands. It focuses on the explanation of the soil behavior mechanisms due to the desiccation and flora-soil interaction phenomena and thanks to the fluviomarine origin of the sediments, on the water to salt stress behavior.

## 2. Material and Methods

### 2.1. Material and Geological Setting

The results presented in this work were obtained from the wetlands located along the Atlantic coast of France. Two areas were studied: the “Marais Poitevin" and the “Marais de Rochefort.” In the two wetlands, the soils are formed on clay dominant sediments named “bri” which result from the silting of an erosion basin in Jurassic limestones during the Flandrian transgression. The ages of the sediments range from 8,000 years to nowadays. In the “Marais Poitevin,” they were deposited directly on granitic rocks on the north part of the basin (Vendée department) and on Jurassic limestones in the south part of the basin (Deux Sèvres and Charente Maritime departments). In the “Marais de Rochefort” they were deposited on the Jurassic limestones. The sediments have fluviomarine origin and were deposited up to +3 m asl which was the maximum level reached by the sea during this period. The origin of the sediments allows the distinction between the marine bri, deposited by sea water, and the continental bri stemming from the peripheral hills. The continental sediments (continental bri) are enriched in organic matter and frequently include peat layers.

The marshes have emerged and dried since at least the Xth century following the sea regression. The polder works started during the XIth century and were the most effective during the XVIIth century. The polder reclaiming was led in order to isolate the marshlands from the sea water inlet along the Atlantic coast and from the continental fresh water inlet along the peripheral hills. Finally the basin was divided into three characteristic territories:

- the “mizottes” which are salt territories lining the Atlantic coast and which are recovered by the sea during high tide,
- the “dry marsh” constituted by the oldest polders which are isolated from the “mizottes” and from the continental water inlet by series of embankments,
- the “wet marsh” located along the periphery of the basin whose role is the storage of rain and fresh water flowing from the peripheral hills.

The climate is coastal-oceanic with a mean annual rainfall of 780 mm distributed throughout two marked seasons: 52% of the rainfall occurs between October and January. The mean June–August temperature and potential evapotranspiration (ETP) from 1971 to 2001 were 16.3°C and 321 mm, respectively [16].

The studies particularly focused on the soils formed on bri in the “dry” and “wet” marshes, excluding the continental organic bri and peat-rich formations. In these “dry” and “wet” marshes the bri shows quite homogeneous mineralogy and texture [9, 14]:

- clay to silty clay texture with 85 to 95% of particles lower than 20 *µ*m and 40 to 60% of particles lower than 2 *µ*m,
- small organic matter content, 0.4 to 2.4 weight %,
- dominant illite, plus kaolinite and illite/smectite mixed layers, and small amount of pure smectite assemblages,
- the measured CEC accord to the illite domain (20–30 meq/100 g),
- the shrinkage, plasticity, and liquidity limits are 20%, 40%, and 70% in gravimetric water content, respectively.

In the 0-to-20 cm soil surface and in the recognized paleosoils the infra 2 *µ*m fraction decreases from the 40–60% of the primary sediment to 5–23%. In parallel the illite/smectite mixed layer assemblages present a weak increase of the smectitic layers % in depth [9, 17]. This increase of smectitic layer % was identified by X-ray diffraction only on the 0.2 *µ*m fraction. Similar mineralogical evolution was identified according to the ages of soils sampled in the successive polders [18]. The phenomenon was attributed to a smectite-to-illite conversion associated to the time of soil maturation.

The general structure of the soils and/or sediment-to-soil evolution was shown by Bernard [9], Bernard et al. [10], and Dudoignon et al. [12]. The authors demonstrated the role of the shrinkage, plasticity, and liquidity limits (*W*_*s*_, *W*_*p*_, and *W*_*l*_, resp.) on the soil structure behavior. In fact the gravimetric water content (*W*) profiles exhibit a progressive *W* increase from the surface to the depth. They can be divided in two superimposed “layers” (Figure 2):

- a *W*_*s*_-*W*_*p*_ surface layer developed down to a level equivalent to *W*_*p*_. The *W* profiles evolve according to the climatic changes and associated wetting-desiccation cycles of surface. The *W*_*p*_ level remains quite invariant through the seasons,
- a *W*_*p*_-*W*_*l*_ subjacent layer virtually insensitive to the seasonal effects and surface desiccation.

This vertical superimposition operates hydraulically as two superimposed media isolated by the *W*_*p*_ layer. The surface layer is a double hydraulic conductivity medium: that is, small permeability of the clay matrix constituting the inner part of prisms and peds (micro-to-centimeter scale) and large permeability of the shrinkage fracture network (macroscopic scale). The subjacent layer is characterized by only the small permeability of the clay matrix which is governed by the microstructure and the particle arrangements in the *W*_*p*_-*W*_*l*_ saturated domain: about 10^−6^ m·s^−1^ in *W*_*l*_ state down to 10^−10^ m·s^−1^ in the *W*_*p*_ state. The *W*_*p*_ layer works as an impermeable line preventing all water transfers from the surface layer down to the subjacent one. The first consequent question is as follows: is the two-layer structure responsible of two superimposed water tables: a surface and free water table fed by the rainwater and a subjacent captive water table resulting from the initial capture of seawater during the sedimentation? In these conditions the water table level descending should involve the descending of the “impermeable” *W*_*p*_ level and the thickness of the surface layer due to eventual leaching by the rain water. The second question is as follows: can there be a vertical fresh-to-sea water exchange from the surface to the subjacent layer? In such conditions the plant growing and the pedodiversity would be mainly governed by the water table level via the eventual addition of a salt stress on the water stress.

**Figure 3:**
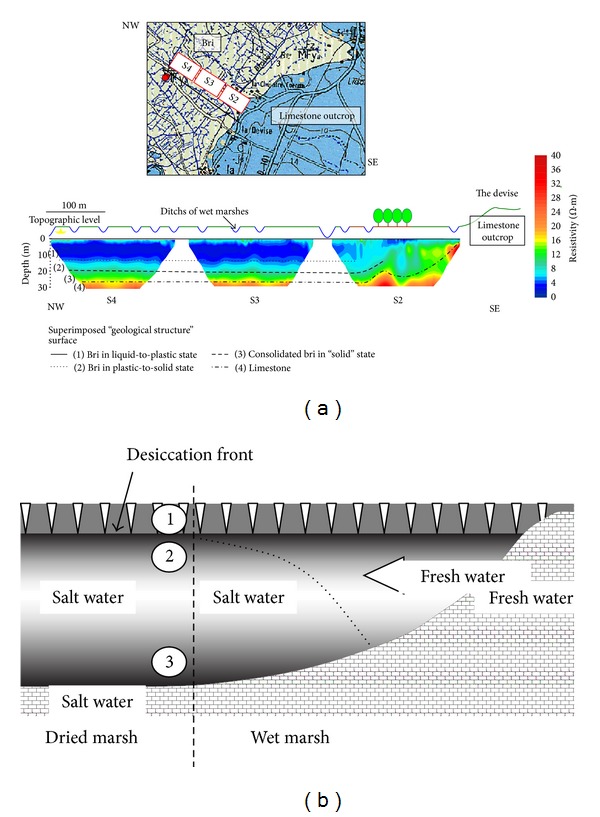
(a) Typical structure revealed by the long resistivity sections in geoelectrical prospecting, (b) schematic representation of the bri structure from the peripheral limestone contact to the inner “dry marsh.” (1) Surface layer impacted by the desiccation-shrinkage effects, (2) *W*_*p*_ to *W*_*l*_ evolution due to the reclaiming and desiccation effect, and (3) *W*_*l*_ to *W*_*p*_ consolidation in depth.

The geological structure of the Marais Poitevin basin was studied by geoelectrical prospecting. The resistivity sections were recorded on twenty areas distributed over the whole basin, from the thin peripheral bri deposits of the “wet” marshes to the inner areas of the “dry” marshes where the bri deposits can reach 30 meter thick. The resistivity sections were obtained using a Syscal R1+ resistivimeter interfaced with a 48 electrodes switch. From the loading to the inverse calculation of the apparent resistivity sections the ELECTRE II, PROSYS, and RES2DINV software were used, respectively. The length of the device around 240 m allows investigations down to 30 meter depth. In these conditions the resistivity sections propose good images of the geological structure with (1) the bri/limestone interface and (2) the typical vertical structure of the bri which can be observed on the whole territory. From the surface down to the limestone contact the bri is characterized by a progressive evolution of its resistivity according to the *W*_*p*_-to-*W*_*l*_ structure evolution and by the consolidation in depth (Figure 3). On the contrary, such long devices miss the subsurface layers (0-to-1 m depth). The resistivity sections show also a horizontally evolution of the bri resistivity from the limestone contact to the “dry” marsh. For equivalent structures the bri is characterized by higher values of resistivity along the limestone contact. It is characteristic of lower water salinities along the peripheral limestone which are indicative of the peripheral fresh water inlet through the bri in *W*_*l*_ “liquid” state.

The 0-to-2 m depth layer was prospected by coupling the resistivity measurement using a salinometer and auger drill holes. The method allowed the measurement of real resistivity associated to measured depth and the calibration of the Archie's law for the clay dominant material constituting the bri [11]:

(1) $$\begin{array}{c} \rho \text{s}=1.01\rho \text{f}\phi^{-2.73}{\text{Sat}}^{-2}, \end{array}$$

with *ρs* is the soil resistivity, 1.01 the formation factor *α*, *ρ*f the water resistivity directly dependent of its salinity, *ϕ* the porosity, −2.73 the cementation factor *m*, Sat the saturation index, and −2 the *n* factor characteristic of the medium.

In order to take into account the clay mineral surface conductivity, the soil resistivity was calculated following the Waxmann and Smits equation [19]. The calculations made with an average 25 meq/100 g CEC and fluid conductivities measured on water sampled in piezometers (2 S·m^−1^ and 4 S·m^−1^ in corn field and grassland, resp.) give results equivalent to the resistivities calculated following the simple Archie's law [15]. In these salt media of coast marshes, the high fluid conductivities minimize the clay mineral surface effect.

**Figure 4:**
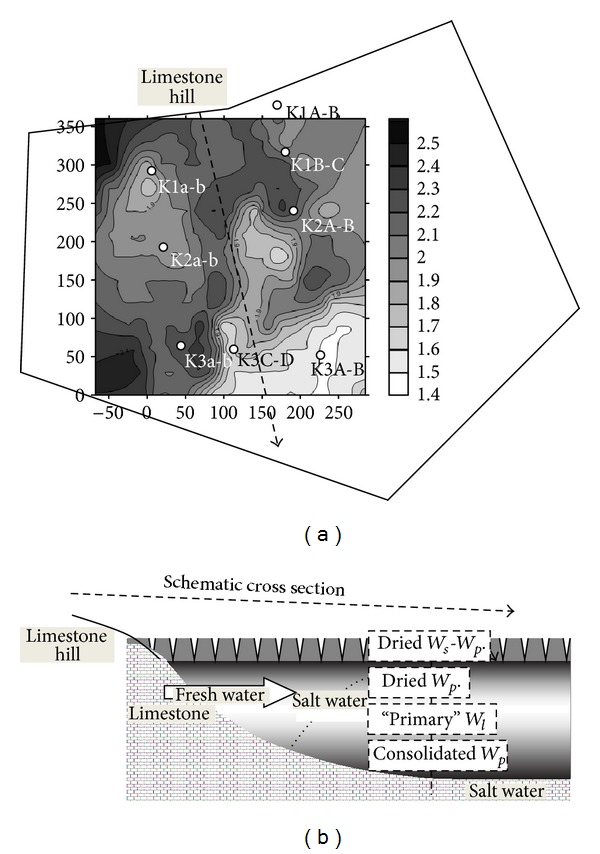
(a) Topographic map (2.5 m altitude near the limestone hill to 1.4 m altitude on K3A-B plot) of the cultivated field with location of the monitoring plots K1 to K3. (b) Schematic cross-section. *W*_*s*_ = shrinkage limit; *W*_*p*_ = plasticity limit; *W*_*l*_ = liquidity limit. Dried = *W*_*l*_-to-*W*_*s*_ behavior due to the surface desiccation; “primary” = initial liquid state; consolidated = *W*_*l*_-to-*W*_*p*_ behavior due to the earth weight.

After a large investigation through the Marais Poitevin territories the study focused on the INRA experimental site of the Saint Laurent-de-la-Prée in the Marais de Rochefort. The main objective is to quantify the role of the water table level variations and rain water-salt water transfer on the plant growing and yields. The studied plots have been distributed on undrained grasslands (since at least 1962) and drained cultivated parcels. The cultivated parcels are lean against the surrounding limestone hill which is the main source of fresh water inlet in the clay-dominant bri system (Figure 4).

The study of the soil structure-hydromechanical property relationships was performed by coupling the water profiles with cone resistance and shear stress profiles [14, 15]. Finally authors used the shrinkage curve of the clay dominant soil to represent the results in successive crossed diagrams [9–15].

### 2.2. Methods

The subsurface soil structure is governed by the desiccation/shrinkage and rehydration/swelling cycles which operate during the seasons. The shrinkage is a 3D mechanism which induces the clay matrix compaction and the opening of a fracture network according to the descending of the desiccation front (Figures 2 and 3). The role of the *W*_*p*_ level was demonstrated by Bernard [9] from the study of the soil structure profiles. The author observed the stop of the shrinkage fractures at the *W*_*p*_ depth and measured the very small hydraulic conductivity of the clay matrix by the oedometer compressibility test.

Two types of *in situ* investigations focused on the eventual water table superimposition and salt-to-fresh geochemical nature of the underground water. The first investigation method was the geoelectrical prospecting. Based on the Archie's law, the apparent soil resistivity gives indications on the salinity of water. The second investigation consists in drilling couple of piezometers in each spot. Each couple of piezometer includes one short piezometer drilled down to 1.50–2.00 meter depth and one long piezometer drilled down to 3 or 6 meter depths. They have been drilled in order to follow the levels of the eventual two water tables and in order to sample the associated groundwaters for chemical analyses. The chemical analyses have been performed on pumped water in each piezometer. They were made by ionic chromatography in liquid phases for Cl^−^ and SO_4_^2−^ anions and by I C P for Ca^++^, Na^+^, K^+^,and Mg^++^ cations (Table 2). Unfortunately, the chemical analyses give global and average chemical compositions of the pumped water which are too inaccurate due to an eventual vertical salinity gradient. In these conditions, the soil profiles were characterized by electrical conductivity (CE_1/5_) measurements. The measurements were made on soils sampled from the surface to 2.00 m depth every 10 cm, according to the clay auger sampling for the water content measurements. The CE_1/5_ was measured on the 1/5 extracts (10 g of dried soil in 50 g of distilled water) [6]. In fact, the CE_1/5_ measurements are made from 10 g of dried soil; thus the CE_1/5_ is independent from the soil water content. Nevertheless it is possible to calculate the fluid conductivity (CEf) for each soil sample following the Montoroi [20] formulae, and Montoroi's equation:

(2) $$\begin{array}{c} \text{CEf}={\text{CE}}_{1/5}\left(5W\right), \end{array}$$

with CEf = the *“in situ”* fluid conductivity, CE_1/5_ the soil conductivity measured in laboratory, and *W* the gravimetric water content.

The study of the soil structure-crop yield relationship was based on the comparison between the CE_1/5_ profiles, the crop yield components, tensiometer profiles recording, and available water capacity (RU) calculations. The yield components included the foot plant number/m^2^, ears/m^2^, depth of rooting, weight of thousand grains, and final yield. Only the final yield in 10^2^ kg/ha is used in the paper. The different yield components were counted in the K1a-b, K2a-b, and K3a-b spots in 2006 and 2007. They were counted in K1A-B, K2A-B, K3A-B, and K3C-D spots from 2008 to 2011. Each spot is 4.5 m^2^ area which corresponds, for example, to 2 contiguous 3 m length rows of corn. In addition the eight K-spots were equipped with tensiometers at the depths of 30, 60, and 90 cm in cultivated field and 40, 60, and 80 cm in the grassland. The suction pressures were measured in 2011 and 2012 in grassland and in a field alternatively farmed for wheat (2011) and corn (2012). The tensiometers are SMS 2500S type. They are limited to 850 mb pressure equivalent to 2.8 pF. Nevertheless they allow the monitoring of the desiccation profiles during the seasons and associated descending rooting.

## 3. Results 

### 3.1. Water Table Levels and Water Chemistry

All the resistivity sections obtained by long devices across the marshes of the Marais Poitevin and Marais de Rochefort accord to the different drill holes made in these territories to identify the same vertical geological structure [9, 21]:

- bri in solid-to-plastic state in surface,
- bri in liquid state approximately between 2 and 5 meter depth,
- consolidated bri in plastic-to-solid state in depth and often evolving to marls,
- subjacent limestone.

Because of the consolidated bri or marl layer directly superimposed on the limestone no significant water transfer was measured between the two formations [21]. Nevertheless the pumped water near the interface presents chemical compositions characteristic of sea water plus fresh water mixture. In fact, from isotopic data, Anongba [21] dated the fresh plus sea water mixture as a phenomenon synchronous of the progressive marine transgressions. The mechanism of fresh water inlet still operates nowadays along the peripheral limestone-bri contact (Figure 3).

**Figure 5:**
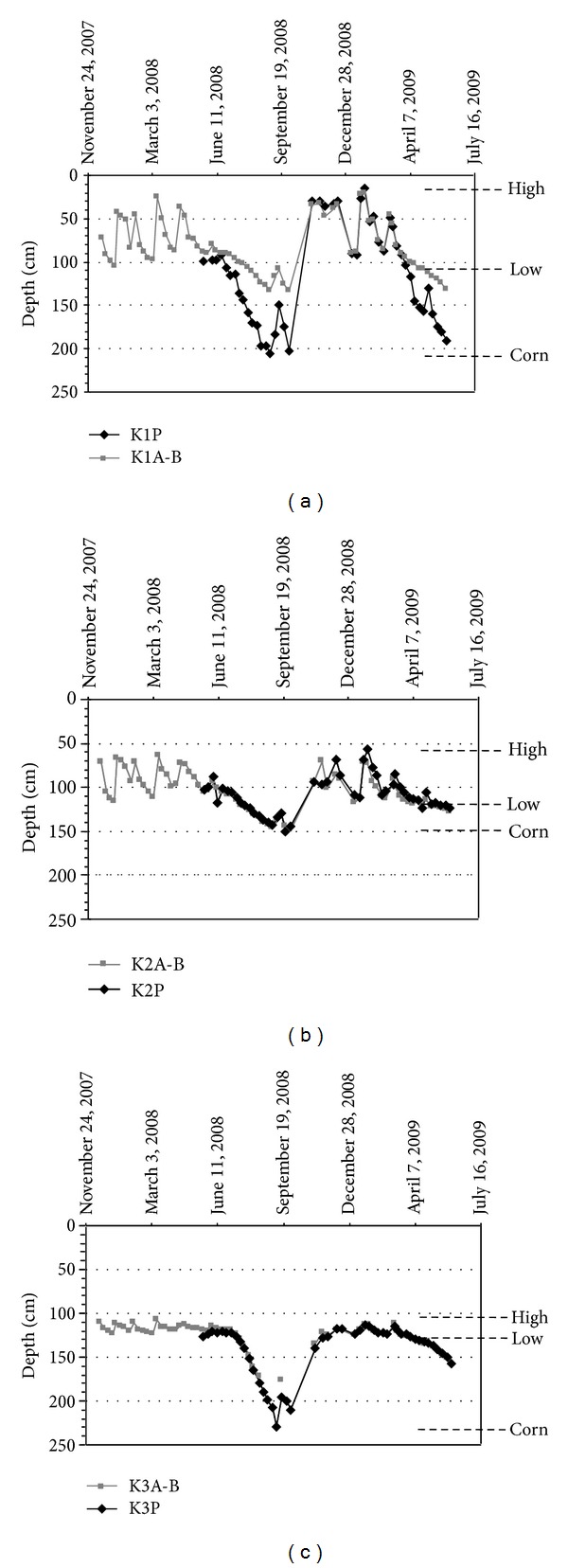
Monitoring of the water table levels in the short piezometers K1A-B (2 m), K2A-B (2 m), and K3A-B (2 m) and in the long ones K1P (3 m), K2P (6 m), and K3P (6 m) from 24 October 2007 to 16 July 2008. High and low = natural water table variation; corn = descending of the water table due to the corn water consumption.

 The vertical fresh water transfers from the fractured bri in solid state of surface, down to the subjacent plastic-to-liquid bri which is initially saturated by salt water, are confronted to the very low permeability of the intermediate *W*_*p*_ level (Figure 2). Nevertheless the monitoring of the water table levels in the couple of piezometers drilled in K1A-B, K2A-B, and K3A-B show only one continuous water table in the bri (Figure 5). A difference between the water table levels measured in a couple of piezometers was only observed in K1A-B during the corn growing. It can be effectively explained by a larger permeability in depth.

**Figure 6:**
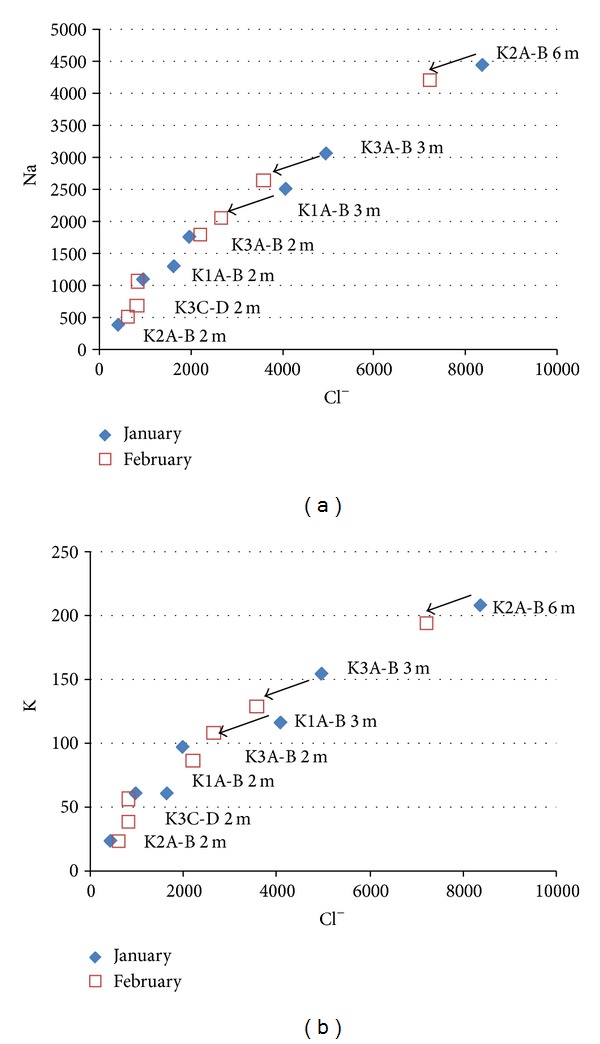
Na and K contents versus Cl^−^ contents (in mg/L) for the different chemical compositions of the waters pumped in January and February 2009 in the piezometers K1A-B (2.00 and 3.00 m), K2A-B (2.00 and 6.00 m), K3A-B (2.00 and 3.00 m), and K3C-D (2.00).

The waters pumped in the double piezometer have been analyzed in January and February 2009. The chemical compositions are characteristic of salt plus fresh water mixtures (Figure 6; Table 1). The low salinities have been measured in the short piezometers (2 m depth), and the high salinities have been measured in the long piezometers (3–6 m depth).

### 3.2. CE_1/5_ Profiles

**Figure 7:**
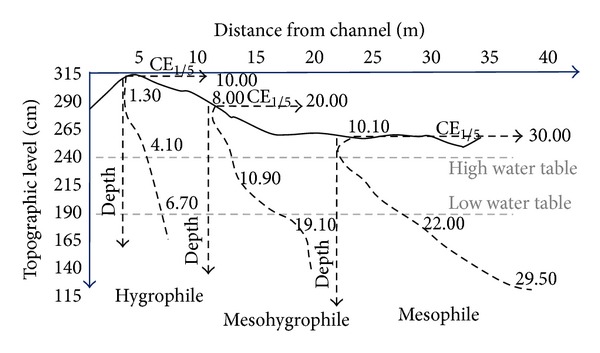
Evolution of the CE_1/5_ profiles according to the topographic level and thickness of leached soil surface (undrained grassland). The mound is due to sediment and soil deposited during the successive channel cleaning works. Low and high water tables indicate the domain of water table variation during the seasons.

The calibration of the Archie's law in these sediments allows the calculation and imaging of the water salinity. Nevertheless the measured resistivity sections are always composed of apparent resistivity and apparent depth. The second “defect” specific to the long geo-electrical device is the lack of data in the 50-to-70 cm surface layer. If the method allows realistic basement recognition, it does not give sufficiently accurate data on the soil sodicity and nape salinity in the 0-to-2 m depth. This lack of data was supplied by the CE_1/5_ profile measurements. This CE_1/5_ profile evolution is well showed in the undrained grassland (Figure 7). For horizontal water tables the profiles are governed by the surface topography and by the channel proximity: from the surface down to 1.50 m depth the ranges of CE_1/5_ evolution are 1–7, 8–20, and 10–30 mS·m^−1^ for the locations close by the channel (5 m), intermediate (10 m), and far-off (22 m), respectively. The three profiles correspond to mesophile, mesohygrophile, and hygrophile systems, respectively [5].

**Figure 8:**
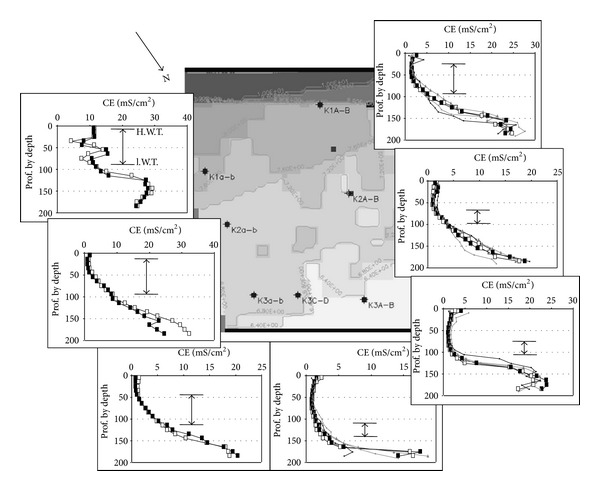
Representation of the CE_1/5_ profiles measured in K1a-b, K1A-B, K2a-b, K2A-B, K3a-b, K3A-B, and K3C-D. The dates of measurements are September 2006 and April 2007 in K1a-b, K2a-b, and K3a-b. They are September 2006, April 2007, December 2007, and June 2008 in K1A-B, K2A-B, K3A-B and, K3C-D. The double arrows indicate the water table variation between the high levels (H.W.T.) and the low levels (l.W.T.).

Similar evolutions have been measured in the cultivated and drained parcel (Figure 8). The CE_1/5_ profiles show progressive increase of CE_1/5_ with depth nearby the limestone hill. The profile patterns evolve with the distance from the limestone hill toward “two slope patterns”: that is, a quite constant small conductivity from the surface to the high water table level and a sharp CE_1/5_ increase in depth. The “two slope pattern” of the CE_1/5_ profiles is enhanced by the gradually descending of the water table as the distance from the hill increases. The CE_1/5_ profiles evolve according to the depth of the water table and consequently according to the thickness of soil surface open to the leaching by rain water (Figures 7 and 8). The CE_1/5_ profiles are also governed by the eventual fresh water inlet from the peripheral limestone and/or from nearby water channels.

**Figure 9:**
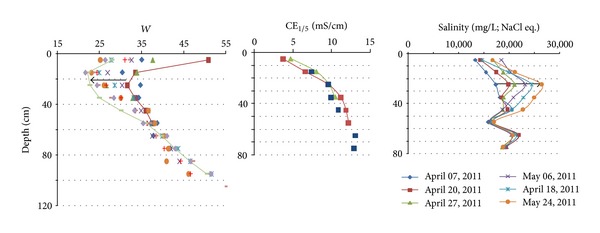
Example of the evolution of the salinity profiles associated with the measured *W* profiles (in grassland). The salinity profiles are calculated in NaCl equivalent, from 07 April to 24 May 2011.

The CE_1/5_ profiles are quite invariant through the seasons. They can be used as references for the calculation of the water salinity profiles. They are deduced from the fluid conductivity (CEf) calculated from the CE_1/5_ and *W* profiles using the Montoroi [20] formulae (Figure 9).

### 3.3. Soil-Plant Interaction

The evolutions of the crop yields on the different parcels are impacted by the prevailing water conditions and the CE_1/5_ profiles. The yields have been often estimated from the depth of the sodic zone [6]. This depth of the sodic zone can be easy to be determined from the “two slope” patterns CE_1/5_ profiles similar to the K3 profiles (Figure 8). On the contrary, it becomes difficult to determine a depth of sodic zone in progressive CE_1/5_ profiles similar to the K1 and K2 profiles (Figure 8). In fact the CE_1/5_ profiles exhibit two superimposed patterns:

- from the surface to the top of the salt water table, the CE_1/5  _values increase following an exponential way
- and from the top of the salt nape to the depth, the CE_1/5  _values show a low increase or remain almost constant.

**Figure 10:**
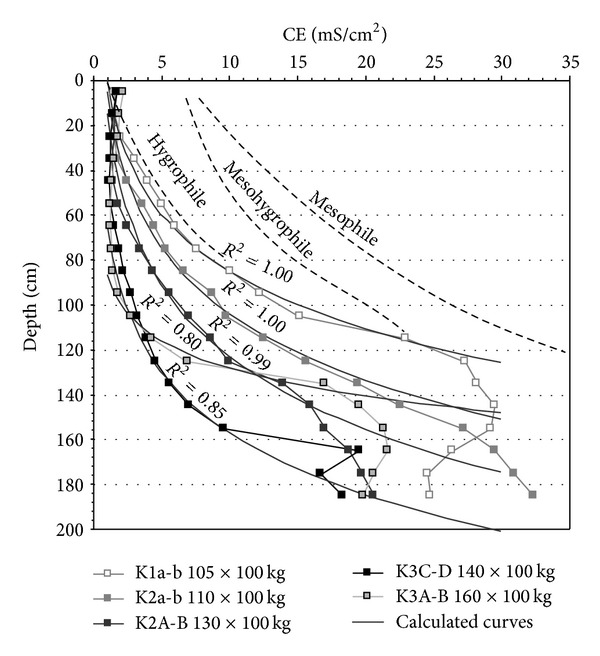
Fits of the calculated CE_1/5_ patterns on the measured profiles from the surface down to the top of salt water table and associated yields in ×100 kg (2006 corn yields for K1a-b and K2a-b; 2008 corn yields for K2A-B, K3A-B, and K3C-D). The corn yields increase progressively from K1a-b to K3A-B following the increasing “slope” of the CE_1/5  _ profiles. Dashed line = domains of mesophile to mesohygrophile and hygrophile CE_1/5_ profiles developed under the grassland.

Whatever are the CE_1/5  _profiles, their upper patterns can be calculated following a simple logarithmic function (Figure 10):

(3) $$\begin{array}{c} D=a*\ln\left({\text{CE}}_{1/5}\right)+b, \end{array}$$

with *D* the depth in m, CE_1/5_ the electrical conductivity in mS/cm^2^, and *a* and *b* two parameters fit to obtain the good superimposition of the calculated CE_1/5_ pattern on the measured CE_1/5_ pattern [14]. Reciprocally the CE_1/5_ profiles can be calculated as follows:

(4) $$\begin{array}{c} \text{CE}=\exp\left(\frac{D-b}{a}\right). \end{array}$$

The shape of the CE_1/5_ patterns is governed by the water table level fluctuations during the seasonal cycles. The deep descending of the water table during the dry season provokes the descending of the desiccation front. The consequences are the leaching of the unsaturated surface layer by the rainfall and a possible phenomenon of capillary ascent. Finally the calculated CE_1/5_ profiles have been related to the corn yields for 2006, 2008, and 2010 [14] (Table 2; Figure 10).

**Figure 11:**
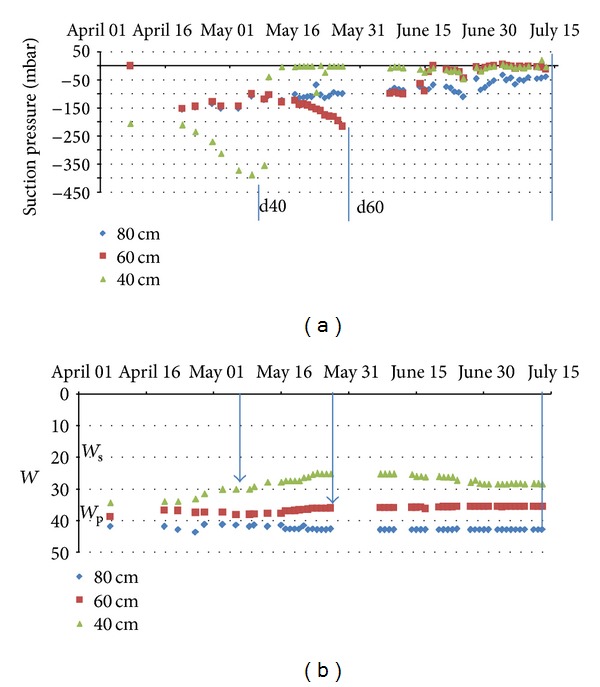
Evolution of the tensiometer pressures and gravimetric water contents at 40, 60, and 80 cm depth from 01 April 2011 to 15 July 2011 in the grassland. The d40 and d60 indicate the disconnection of the porous plugs at the depths of 40 and 60 cm, respectively.

**Figure 12:**
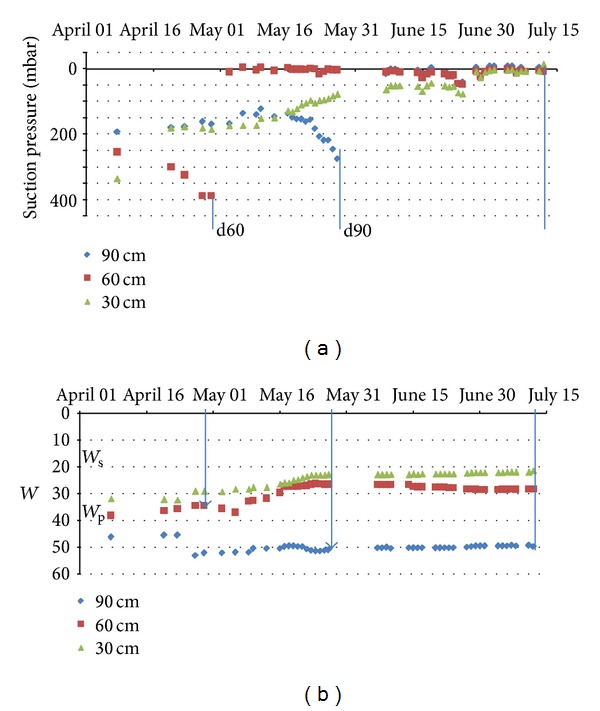
Evolution of the tensiometer pressures and gravimetric water contents at 30, 60, and 90 cm depth from 01 April 2011 to 15 July 2011 (K2A-B; wheat field). The d60 and d90 indicate the disconnection of the porous plug at the depths of 60 and 90 cm, respectively.

The descending of the desiccation front is caused by the summer climatic conditions enhanced by the water consumption by the plants. The interaction between the water profiles and the plant growing can be suggested by the evolution of the tensiometer profiles (Figures 11 and 12).

In the grassland the progression of the desiccation front is well shown by the “increase” of the measured suction pressures and by the time offset between the 40 cm and 60 cm measured pressures. The low suction pressures measured at the depth of 80 cm are due to the near water table level in this undrained field. Nevertheless, the d40 and d60 plug disconnections operate at different gravimetric water contents and thus at different suction pressures. In these conditions the plug disconnection cannot be explained by a simple difference between the gravimetric water contents.

In the drained wheat field a similar time offset is observed between the suction pressures measured at the depths of 60 and 90 cm. The d60 and d90 plug disconnections operate also at different water contents. The mechanism is similar to the mechanism observed in the grassland. Two main differences emerge: the deepening of the desiccation front in this drained field and the behavior of the tensiometer measurements near the surface (30 cm). The surface desiccation does not imply any increase of the measured suction pressures at the depth of 30 cm. In fact the 0–30 cm surface layer is affected by the tillage every year. The consequence is the formation of a “crumble” soil structure in surface characterized by a large unsaturated meso- to macroporosity. At variance the soil structure in depth, unaffected by the tillage, evolves according only to the clay matrix microstructure along its shrinkage curve.

**Figure 13:**
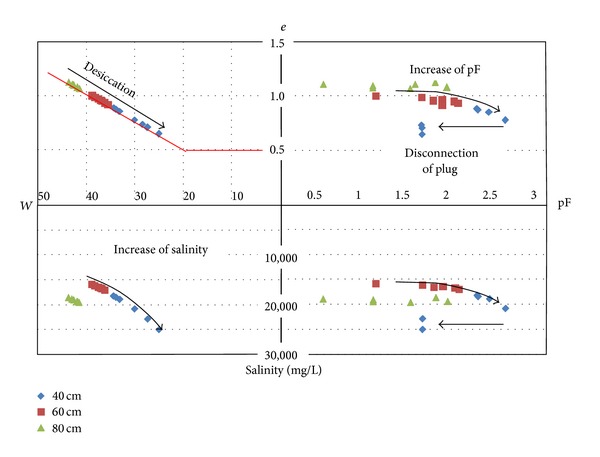
Example of *W*-*e*-pF-Salinity relationship (grassland). The shrinkage effect (*W*-*e*) causes the increase of the tensiometer pressure and associated pF (*e*-pF). The shrinkage fracture opening causes the disconnection of the porous plug. The salinity behavior can be represented due to the water content and tensiometer values.

Using the CE_1/5_ profile of each spot as reference, the water salinity may be calculated for the successive depth using the couple water content-CE_1/5_ following the Monteroi equation. For each depth the suction pressure accords to the water content. In these conditions the clay matrix shrinkage curve can be used as a tool to represent the parallel evolution of the water content, tensiometer pressure, and water salinity (Figure 13).

### 3.4. Available Water Capacity Behavior

The two main parameters which act on the plant growing and flora diversification are the water and salt stresses. The high water salinities limit the plant growing to the mesophile systems: the moderate water consumption limits the salt “storage” in the plant organism. On the contrary the poor water salinity allows the growing of hygrophile plants: despite the high water consumption the salt “storage” remains limited in the plant organism. In these coastal marshlands the two water and salt stresses are governed by the mechanisms of “wet land” to “dried land” conversion:

- the water table descending caused by the hydraulic works and associated surface desiccation,
- the soil structure behavior due to the shrinkage properties of the clay dominant material,
- the eventual fresh water inlet from channel of peripheral limestone hill,
- and, consequently, the progressive evolution from the mesophile systems to mesohygrophile and hygrophile ones.

Considering the water table level, the plant growing and the root deepening are confronted firstly to the available water capacity (RU) of the unsaturated soil and secondly to the level of anaerobic in the subjacent saturated medium. The high water table level prevailing during the wet winter season stops the plant rooting. It is the main problem for the yields of the winter cultivation as wheat. The quite linear 1/1 water table depth/root depth ratio was demonstrated in these marshes by Pons et al. (2000) [1]. The problem of rooting depth has been in part resolved in the 70–80 years by the extensive politic of drainage. On the salinity point of view, the CE_1/5_ profiles indicate low salinities near the surface and high salinities in depth. The problem becomes evident for the yields of the summer cultivation as corn. Due to the drastic decrease of the available water capacity of soil in surface the plant rooting has to progress in depth and reaches the salt levels.

The available water capacity is usually calculated according to the soil texture and/or according to the rainfall-evapotranspiration balances [22]. The available water capacity (RU) can be calculated as follows:

(5) $$\begin{array}{c} \text{RU}=h\times d\left(W_{\text{fc}}-W_{\text{wp}}\right)\times 10, \end{array}$$

with *h* the thickness in m, *d* the apparent density, *W*_fc_ the water content at field capacity, and *W*_wp_ the water content at wilting point.

The *W*_wp_ is usually assessed as the equivalent 4.2 pF (15,000 hPa tensiometer pressure), thus around the *W*_*s*_ for the clay dominant soils. The difficulty is the choice of the *W*_fc_ equivalent to the 2.5 pF (330 hPa tensiometer pressure) which is “located” between the *W*_*s*_ and *W*_*p*_ of the clay dominant soils. The *W*_fc_ is mainly governed by the soil texture: that is, from 29% to 32% for clay-dominant soil to silty clay soils [23]. The *W*_fc_ and *W*_wp_ may be calculated taking into account the sand %, clay %, and organic matter % (OM) as follows [24]:

(6) Wfc=257.6−(2∗sand%) +  (3.6∗clay%)+(29.6∗OM%),Wwp=26+(5∗clay%) +(15.8∗OM%).

Thus the RU may be calculated for each soil layer as follows:

(7) $$\begin{array}{c} \text{RU}=\left(W_{\text{fc}}-W_{\text{wp}}\right)*h, \end{array}$$

with RU in mm and *h* the layer thickness in m.

**Figure 14:**
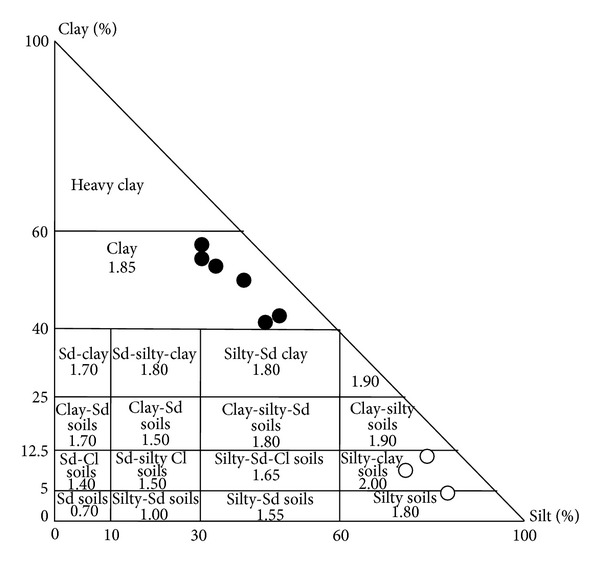
Location of the soil texture and associated available water capacity RU. Black circles = clay dominant sediment to soil; white circles = silty soil surface and paleosol identified on the INRA St Laurent experimental site.

According to their textures the soils developed from the bri exhibit two groups of RU values (Figure 14):

- the sediment-to-soil transition characterized by nonsignificant textural change. The material is composed of clay dominant material. The RU remains around 1.80 mm,
- the soil surface and the paleosol characterized by silty clay soil texture. The RU remains around 1.80–2.00 mm.

**Figure 15:**
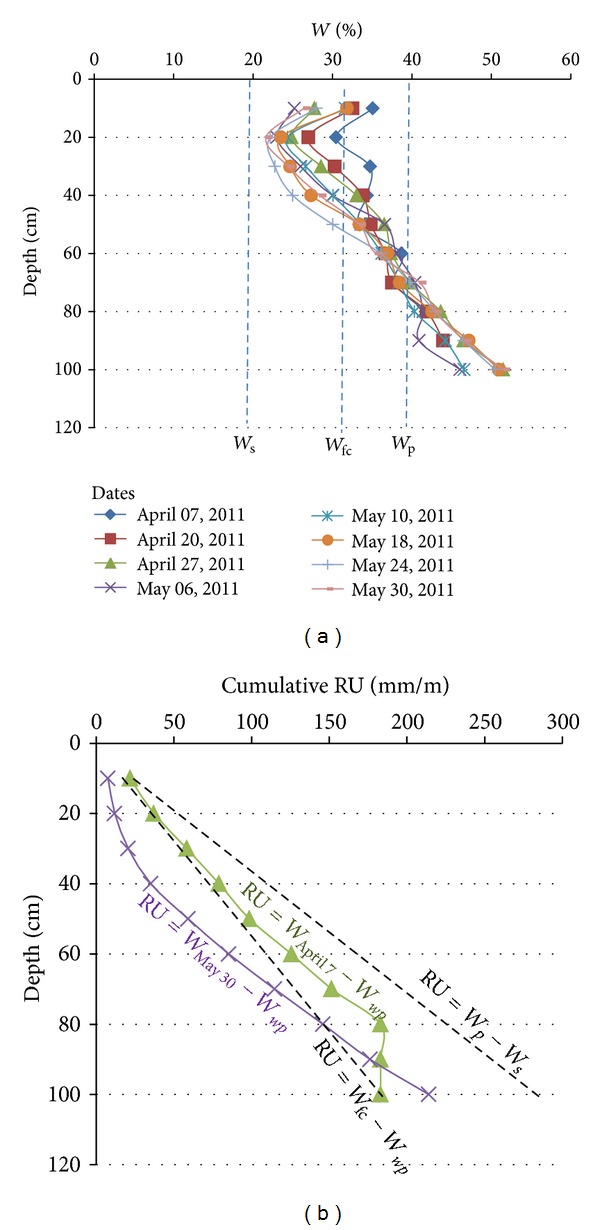
Evolution of the *W* profiles (a), RU profiles, and RUr profiles (b) from 07 April to 30 May in the grassland. *W*_*p*_ = plasticity limit; *W*_*s*_ = shrinkage limit; *W*_wp_ = wilting point (*W*_wp_ = *W*_*s*_); *W*_30/05_ = water profile at 30 May; *W*_07/04_ = water profile at 07 April.

In fact, if the soil may be overall characterized by its RU in mm/m, for a vertical profile, the RU is generally calculated as a cumulative RU which is obtained by the RU (mm/m) × depth for each level. The progressive soil desiccation which progresses from the surface down to the depth may be used to calculate the “real or residual” available water capacity (RUr) available for plants through the season. The real or residual soil RUr profiles may be calculated using the difference between the water profile at the considered date and the reference *W*_fc_ (Figures 14 and 15):

(8) $$\begin{array}{c} \text{RUr}=h\times d\left(W_{7/04}-W_{\text{wp}}\right)\times 10, \end{array}$$

with *h* the thickness in m, *d* the apparent density, *W*_*d*/*m*_ the day/month measured water content at each depth, and *W*_wp_ the water content at wilting point. 

This evolution of the real RUr profiles with time can inform on the soil-root interaction: that is, potential water consumption, wilting approach, and rooting in depth.

**Figure 16:**
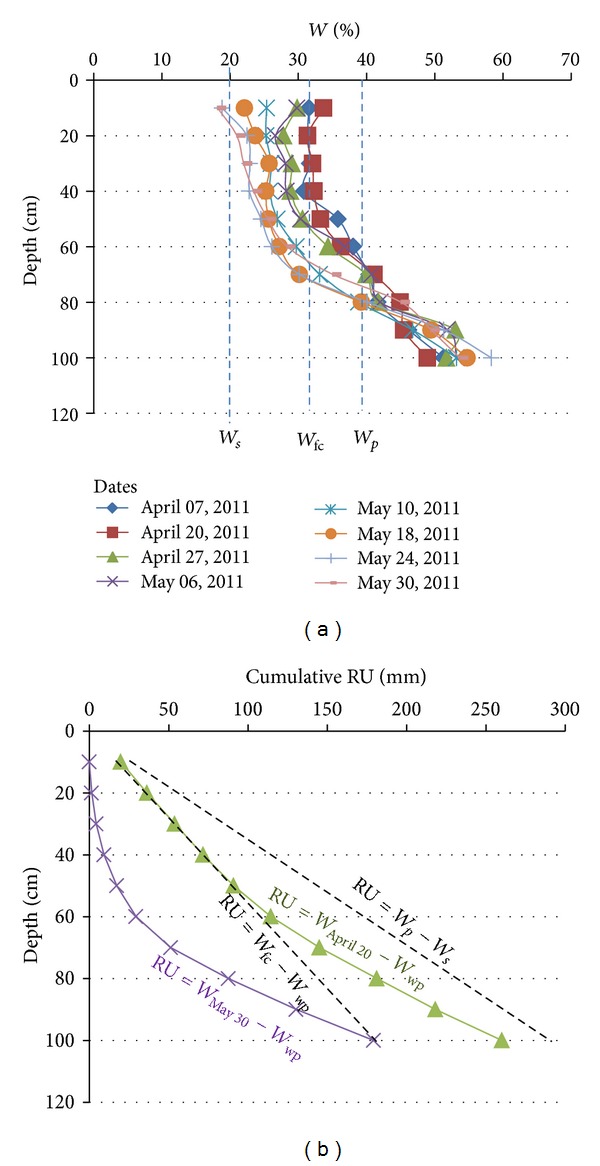
Evolution of the *W* profile (a), RU profiles, and RUr profiles (b) from 20 April to 30 May in the K1A-B under corn farming. *W*_*p*_ = plasticity limit; *W*_*s*_= shrinkage limit; *W*_wp_ = wilting point (*W*_wp_ = *W*_*s*_); *W*_30/05_ = water profile at 30 May; *W*_20/04_ = water profile at 20 April.

**Figure 17:**
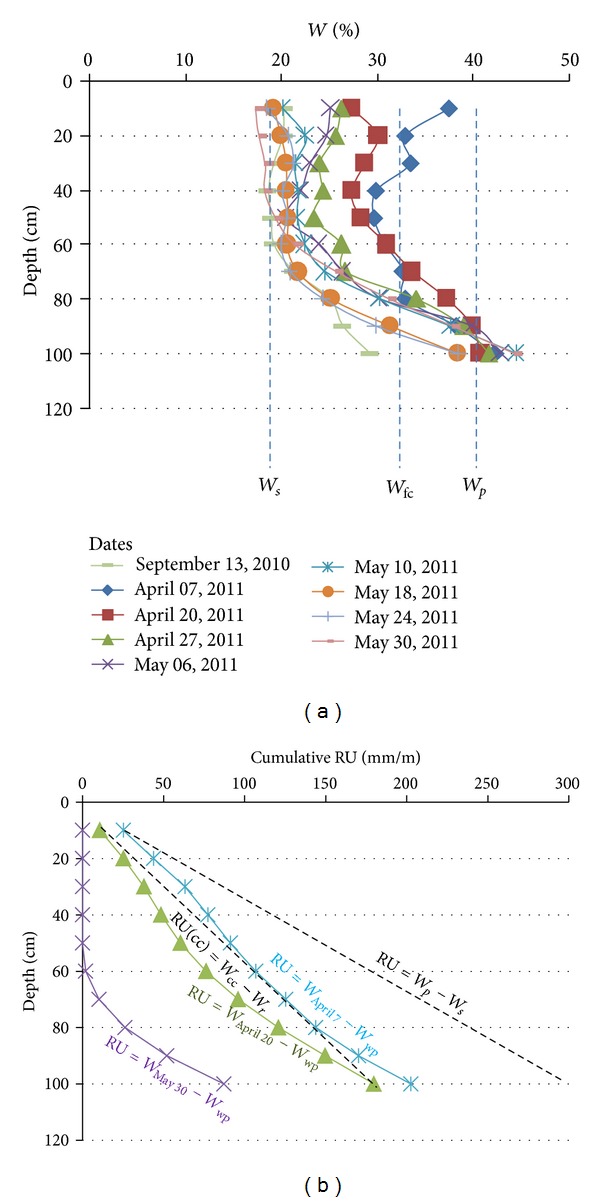
Evolution of the *W* profile (a), RU profiles, and RUr profiles (b) from 07 April to 30 May in the K2A-B spot. *W*_*p*_ = plasticity limit; *W*_*s*_ = shrinkage limit; *W*_wp_= wilting point (*W*_wp_ = *W*_*s*_); *W*_30/05_ = water profile at 30 May; *W*_20/04_ = water profile at 20 April; *W*_07/04_ = water profile at 07 April.

**Figure 18:**
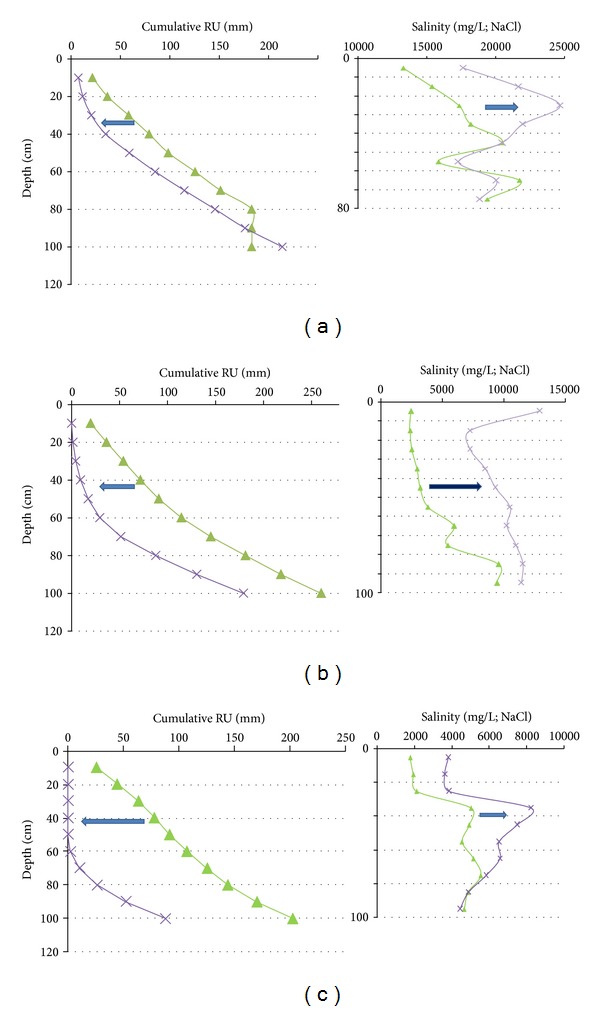
Simultaneous effects of the desiccation on the RUr profiles and on the water salinity. (a) In the grassland the high initial salinity drastically increases during the surface desiccation. (b) In the K1A-B the increases of salinity are moderated by the fresh water inlet from the limestone hill. (c) In the K2A-B the increases of salinity are moderated because of small residual salinity of the leached soil. Triangle = 07 April in K2A-B and grassland profiles and 20 April in K1A-B profiles; crosses = 30 May profiles.

 In the corn field and grassland examples, in April, the RUr profiles calculated by differences between the *W* profiles and the *W*_wp_ are quite superimposed on the theoretical RU = *W*_fc_-*W*_wp_. In May the RUr profiles calculated by differences between the *W* profiles and the *W*_wp_ are largely shifted to the 0 vertical axis (Figures 15, 16, and 17). The shift results from the water evaporation plus plant consumption: that is, synchronous descending of the desiccation front and rooting. On the other hand, in such brackish to salt media and due to the quite stable CE_1/5_ profiles, the soil desiccation implies the increase of the water salinity for each level (Figure 18).

## 4. Discussion

### 4.1. Discrete Mineralogical Evolution

Whatever the authors, the impacts on the mineralogy of the clay dominant sediments have been interpreted as smectite-to-illite conversion associated to the soil maturation in oxidative conditions. The clay mineral evolution was demonstrated by Righi et al. [18] on soil surfaces dated from the ages of the successive polders in the west part of the Marais Poitevin. It was also demonstrated through vertical profiles sampled throughout the West French marshlands by the vertical decrease of the smectite contents from the sediment in depth to the soil surface. It was also verified in paleosols identified between 1 and 2 meters depths [9, 17]. In fact the smectite-to-illite conversion has been observed via XRD patterns performed on the infra 0.02 *µ*m fraction and only thanks to mathematical decomposition of the XRD patterns. The impact of the phenomenon is very weak due to the whole clay dominant soil behavior. This weak vertical decrease of the smectite content in the clay dominant matrix was also explained by a vertical leaching of the very thin particles by rain water. This second argument is based on the associated textural evolution from the surface to the depth. The analyzed soil surface (0–30 cm depth) and paleosol exhibits textures of more silty soils whereas the whole material exhibit clay dominant texture [9, 10]. In fact these very weak mineralogical and textural evolutions are common throughout the territories and do not imply significant differences on the marsh management.

### 4.2. Dominant Role of the Soil Structure Behavior

**Figure 19:**
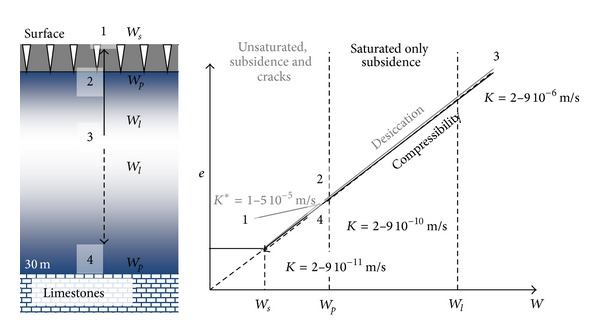
Schematic representation of the relations prevailing between the microstructure behavior of the clay matrix and the macroscopic behavior at the field scale during the surface desiccation phenomenon (3 to 2 to 1) and during the consolidation in depth (3 to 4). *K* = hydraulic conductivity measured by oedometer compressibility test on unfractured and saturated clay matrix K* = surface hydraulic conductivity measured by Porcher test on the fractured soil (from Dudoignon et al. [13]).

Since the Xth century the soil formation in the coastal marshlands is governed by the structural evolution of the primary sediment in surface caused by the desiccation mechanism. In depth, the primary clay dominant sediment, saturated by sea water, is consolidated because of the earth-or-sediment weight pressure. The two mechanisms induce similar structural evolutions of the clay matrix which are provoked by the clay particle rearrangement. In a void ratio (*e*) versus water content (*W*) the desiccation way and the consolidation way are superimposed (Figure 19) [13, 25, 26]. They provoke the outlet of interparticle water and the decrease of the microporosity by rearrangement of the clay particles [18].

Finally, the initial muddy sediment has been affected by two opposite mechanisms:

- the progression of the desiccation-shrinkage front from the surface to the depth, from *W*_*s*_-*W*_*p*_ in surface down to the *W*_*l*_ state,
- the consolidation in depth due to the earth pressure, from the *W*_*l*_ state to the *W*_*p*_ state.

The two mechanisms act to the vertical evolution of the bri in the three-layer structure [10, 12, 13, 15] (Figure 19):

- solid-to-plastic state near the surface characterized by low clay matrix hydraulic conductivity,
- intermediate plastic-to-liquid state, medium hydraulic conductivity,
- plastic-to-solid state in depth, low hydraulic conductivity.

The hydraulic functioning of the marshes is governed by this natural vertical structure caused by the hydraulic developments which have been successively built in order to accelerate the reclaiming and in particular to isolate the wet marshes from the dried marshes. The vertical structure and the very low Wp hydraulic conductivity limit the vertical inlet of rain water. On the contrary it allows the fresh water inlet from the peripheral limestone through the intermediate bri in liquid state.

**Figure 20:**
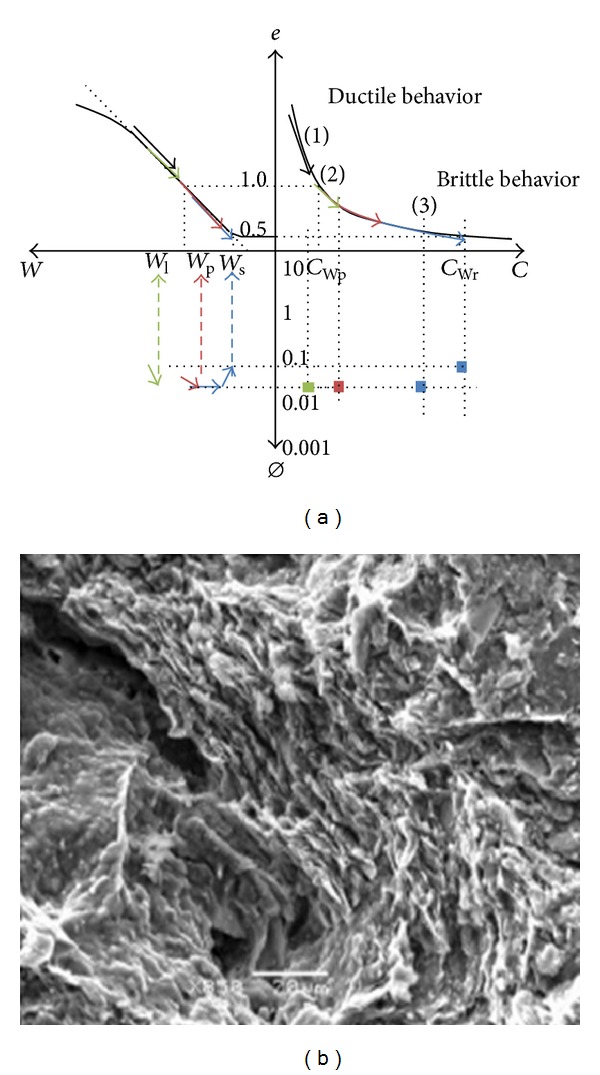
Schematic representation of the mesopore and fracture opening of the clay matrix for the different water contents associated with the increasing depths. (a) Ductile-to-brittle behavior of the clay material taking into account the *W*-*C* couple evolution and the successive steps of fracturing: (1) ductile behavior in the plastic-to-liquid state domain, (2) plasticity limit (*W*_*p*_), (3) brittle comportment in the solid state [14]. *W* = gravimetric water content; *e*= void ratio; *C*= shear stress; *Ø* = equivalent pore diameter. (b) SEM microphotography of a mesopore adjacent to the *W*_*s*_ clay matrix.

The vertical evolution has been recorded on many sites by cone penetrometer, shear stress, bulk density, and water content profiles. Thanks to the textural and mineralogy homogeneity of the clay dominant material, the clay matrix shrinkage curve has been used as tool for the representation of the numerical relationships which can exist between the hydromechanical properties and the microstructure of the clay material [10, 12, 13, 15] (Figure 20). The microstructure-cone resistance (Qd) and microstructure-cohesion (*C*) relationships have been written as Perdok's equation or power law [10, 14, 15]. The same authors have presented the clay matrix microstructure-hydraulic conductivity relationship from simple calculations and oedometer measurements.

### 4.3. Desiccation FrontWater and Salt Stress Relationship

The desiccation, locally accelerated by the drainage, has induced the descending of the water table and the increase of the solid-to-plastic layer thickness from the surface. Because of the high shrinkage properties of the clay material, this drying upper layer was crosscut by the shrinkage fracture network. In these conditions, the mechanism has allowed the surface desalination by the rain water leaching through the unsaturated plastic-to-solid clay material via the fracture network. These drying and desalination mechanisms have been the two main objectives of the reclaiming. Nevertheless the obvious questions are as follows.

- How to characterize the eventual fresh-to-salt water exchange and the soil salinity profiles?
- What are the consequences on the soil-plant relationships?
- Eventually, what are the most suitable tools or methods for mapping the fresh to salt area?

The tensiometer pressure measurements give information about the water content-microstructure couple during the desiccation phenomenon. The measured tensiometer pressures are governed by the reduction of micropores due to the desiccation-shrinkage coupling. The tensiometer pressure-equivalent pore diameter can be calculated via the Jurin law. The disconnection of the porous plug observed at the depths of 40, 60, and 90 cm operates following a time offset which may be interpreted as the descending of the desiccation front (Figures 11 and 12). The clay matrix desiccation induces the clay matrix shrinkage and provokes the additional opening of the mesopores and/or mesofractures (Figure 20(b)). The geometry of the shrinkage fracture network depends on the initial *W*-*C* couple of the clay matrix and shift of the clay matrix from the ductile-to-brittle behavior: that is wide and spaced fractures around the *W*_*l*_ domain and thin and close to each other in the *W*_*p*_-to-*W*_*s*_ domain (Gallier, 2011 [14]; Gallier et al., 2012 [15]; Figure 20). In these marshlands the *W* profiles indicate the *W* increase with depth. Thus, for the successive depths, the mesopore-to-fracture opening operates at different initial hydric states of the clay matrix and different *W*-*C* couples. The differences between the tensiometer pressures associated with the plug disconnection at the different depths can be explained by the vertical evolution of the *W* profiles from *W*_*s*_ in surface to *W*_*l*_: that is, dominant mesoporosity and close mesofractures near the surface and larger and spaced fractures in depth which can open for higher water contents (Figure 20).

The geo-electrical prospection is able to give vertical “images” of the bri structure down to the limestone basement. The Archie's law was calibrated taking into account the porosity and the water resistivity (equivalent salinity) for our clay dominant material [11, 13, 15]. Nevertheless, the surface recording of resistivity sections is limited in accuracy because of the measurement of apparent resistivity for apparent depths. Yet the resistivity-porosity relationship given by the Archie's law allows the transformation of the resistivity section into a permeability section which can be indicative of the local hydraulic running [13]. The most realistic vertical recording of resistivity profile can be obtained by coupling salinometer and water content profiles [11]. In fact the characterization of the profiles is easy by auger sampling which allows the density, water content, and CE_1/5_ measurements for each depth of sampling. The water salinity is easily calculated from the couple *W*-CE_1/5_.

**Figure 21:**
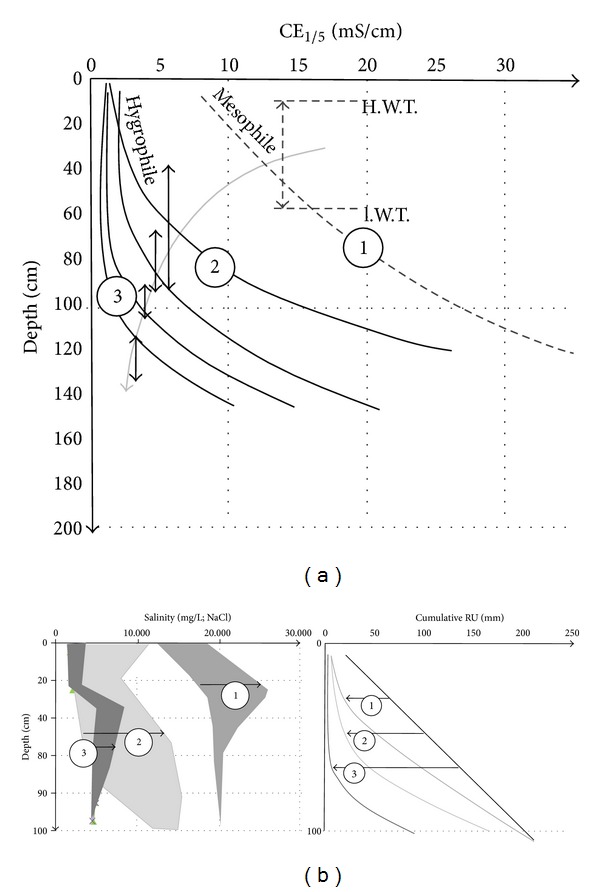
(a) Schematic evolution of the CE_1/5_ profiles induced by the water table descending. Grey arrow = water table trend; double vertical black arrows = water table fluctuation between the high water table level (H.W.T.) and the low water table (l.W.T.). 1 to 2 indicate the transition from the mesophile to hygrophile systems. (b) Parallel evolutions of the RU profiles and salinity profiles characteristic of the three studied systems. Straight black line = initial (textural) cumulative RU profiles. 1, 2, and 3 residual cumulative RUr profiles calculated after the plant growing under the grassland and under the cultivated field (example of wheat). Trend 1 = grassland; trend 2 = cultivated area nearby the limestone hill, thus impacted by the fresh water inlet; trend 3 = cultivated area far from the limestone hill.

The CE_1/5_ profiles have progressively matured due to the descending water table. They are quite insensitive to the season, at least over a few years. Nowadays they represent the result of the effects of desiccation and desalination induced by the successive reclaiming works since the Middle Ages. Two factors act on the CE_1/5_ profile evolution: the depth of the water table quite equivalent to a thickness of soil leaching by the rain water and the eventual fresh water inlets. The mechanism is condensed by the observations made on the undrained grassland: the differences in leached layers are generated by the surface topography considering the water table horizontal, and the water inlet can be observed nearby the channel boundary (Figure 7). The thickness of leaching added to the peripheral fresh water inlet induces the progressive shift of the CE_1/5_ profiles from a “mesophile profile,” characterized by a very high CE_1/5_ values (10–30 mS/cm) and a very weak slope of the profile, to a “hygrophile profile” characterized by low CE_1/5_ (1–7 mS/cm) values and a hard slope of profile near the surface. In fact the comparison between the undrained grassland and the drained cultivated field allows to observe the continuity of CE_1/5_ profile evolution from the undrained to drained domains (Figures 10 and 21(a)). The water table depth impacts directly on the shape of the CE_1/5_ curves: slope evolution from the surface and thickness of leaching. The evolution from the mesophile systems to the hygrophile ones clearly appears in the RUr and associated salinity profiles (Figure 21(b)). For equivalent initial cumulative RU profiles, due to the homogeneous texture, the initial salinity of the hygrophile systems (3 and 2) is restrained to the 2–5 mS/cm domain (Figure 21(a)). For the mesophile system the salinity is between 10 and 20 mS/cm. Finally three types of couple salinity-RUr profiles can be distinguished for the different spots. They are governed by presence or not of drainage, the proximity of the fresh water source, and the difference in plant farming. They describe the soil functioning from the mesophile to hygrophile systems.

The mesophile profile type is characteristic of the undrained territories which are traditionally occupied by cattle, thus let in grassland. The water consumption by the grassland is temperate. Thus the residual RUr profile is weakly shifted from the initial RU line (Figure 21, trend 1). Nevertheless the increase of soil salinity is very high because of the residual salinity due to the lack of leaching.

The hygrophile profile types exhibit two trends: trend 2 corresponds to the nearby limestone profile with fresh water inlet and trend 3 far away the hill. In trend 2, the fresh water inlet allows the high water consumption by the plant. The shift of the residual RUr profile from the initial RU is moderated. The evolution of the salinity between the initial and residual RU profiles is significant. It results from the fresh water plus salt water mixture. In trend 3, the lack of water inlet due to the water consumption by plants induces the high shift of the RU profile from the initial one. The general low water table level is associated with the high thickness of leached layer; thus the initial low salinity limits the increase of salinity during the desiccation period.

## 5. Conclusion

The successive works made on the French Atlantic marshlands in recent years have shown the role of the clay matrix microstructure on the clay dominant soils behavior. The results have been obtained thanks to the mineralogical and textural homogeneity of the initial sediments and thanks to the very low impact of the reclaiming, dated from the Middle Ages, on these two parameters. The sediment-to-soil evolution has mainly been governed by the desiccation-shrinkage mechanisms of the clay matrix. Thus, the soil microstructure-hydromechanical property relationships have been represented in successive clay matrix shrinkage curve-cone resistance or cohesion-hydraulic conductivity crossed diagrams [9–15]. The clay matrix behavior allows the explanation of the hydrologic functioning of the marshlands taking into account the desiccation effect of surface on the clay dominant soils and the compressibility effect on the clay dominant sediment in depth.

The two main objectives of the reclaiming have been the descending of the water table levels and the desalination of soil. Sufficient for extensive grazing grounds, the reclaiming has been completed by the drainage for the cereal cultivation from 1970. The interaction between the water table level and the soil salinity is easy to follow using the CE_1/5_ profiles. These ones are characteristics of the hydraulic history of the territories. Quite invariant during the seasons, they can be explained as the result of the water table management many years ago. Moreover, they can be used to predict the crop yields.

The shift of the study from the grassland to the drained cultivated field allows the presentation of a quantitative evolution of the CE_1/5_ soil profiles from the undrained to the drained lands. Thanks to the Montoroi equation [14, 20] and coupled with the moisture profiles they allow the calculation of the water salinity profiles and their evolutions during the seasons. Thus, the *in situ* tensiometer recordings and calculated water salinity can be represented taking into account the clay matrix microstructure in a *W*-*e*-pF-water salinity crossed diagram.

Finally, the soil-plant interaction can be “modeled” for the grassland and cultivated fields taking into account the location of the studied spots relative to the fresh water inlet from the peripheral limestones. The “model” is based on the shift of the “initial” available water capacity profiles of soil to the “residual” available water capacity profiles resulting from the plant growing and associated shift of the water salinity profiles. The results show the evolutions of the different associated profiles in the mesophile, mesohygrophile, and hygrophile systems from undrained to drained territories.

The method can be used to characterize the available diversity of the plants due to the different domains of water and salt stress on these territories reclaimed on fluviomarine deposits. It can be used as diagnostic tool and/or projection tool for the crop yields but also for the flora evolution due to extension of the “dry” territories against the residual “wet” territories which can be induced by different choices of hydraulic managements or by the climatic changes. In the opposite direction it can be used as projection tools for the evaluation of the eventual “dry” marsh rewetting.

## Figures and Tables

**Table 1:** Chemical compositions (in mg/L) of the waters pumped in January and February 2009 in the piezometers K1A-B (2.00 and 3.00 m), K2A-B (2.00 and 6.00 m), K3A-B (2.00 and 3.00 m), and K3C-D (2.00).

	Cl^−^	Na	Ca	K	Mg	S	SO_4_	Total salinity (mg/L)
January piezometer								
K1A-B (3.0 m)	4080	2508	361.1	116.6	339.4	756	2269	9673.9
K2A-B (6.0 m)	8370	4438	247.5	208.4	355.5	506	1517	15136.5
K3A-B (3.0 m)	4960	3059	301.8	154.8	513.2	1063	3189	12178.2
K1A-B (2.0 m)	1640	1302	330	61.16	199.1	427	1282	4814.16
K2A-B (2.0 m)	430	390	190.5	24.02	66.09	191	573	1673.51
K3A-B (2.0 m)	1980	1760	580.8	97.51	253.5	790	2369	7040.71
K3C-D (2.0 m)	970	1098	594.4	61.2	144.1	527	1580	4447.8

February piezometer								
K1A-B (3.0 m)	2207	1797	292.5	86.59	242.3	543.2	1629.6	6254.99
K2A-B (6.0 m)	7201	4203	230.8	193.7	467.7	455	1365	13661.2
K3A-B (3.0 m)	3578	2643	435.9	128.8	431.4	821	2463	9680.1
K1A-B (2.0 m)	825	683	263.1	38.84	131.3	303.7	911.1	2852.34
K2A-B (2.0 m)	631	514.6	218.7	23.48	76.93	197.5	592.5	2075.21
K3A-B (2.0 m)	2661	2057	448.1	107.7	356.8	837.3	2511.9	8142.5
K3C-D (2.0 m)	834	1068	265.3	56.48	139.2	530.2	1590.6	3953.58

**Table 2:** Culture yields in 10^2^ kg/ha measured in 2006, 2008, and 2010 for corn field and 2007, 2009, and 2011 for wheat field.

	Corn			Wheat		
	2006	2008	2010	2007	2009	2011
K1 a-b	104.4			56.1		
K2 a-b	109.4			67.2		
K3 a-b	143.3			67.1		
K1 A-B		126.9	105.1		92.1	78
K2 A-B		132.8	111		103.8	72
K3 A-B		157.5	117		99.6	97
K3 C-D		147.3	111.6		108.3	86
